# Health professionals’ experiences of rapport during telehealth encounters in community palliative care: An interpretive description study

**DOI:** 10.1177/02692163231172243

**Published:** 2023-05-02

**Authors:** Wendy English, Jackie Robinson, Merryn Gott

**Affiliations:** School of Nursing, University of Auckland, Auckland, New Zealand

**Keywords:** Telehealth, rapport, soft skills, patient-health professional relations, interpersonal relations, families, palliative care, qualitative research

## Abstract

**Background:**

Despite the reported importance of rapport, there are knowledge gaps in the ways rapport is developed and experienced by health professionals during telehealth calls in palliative care.

**Aim:**

To gain an understanding about developing rapport during telehealth calls by exploring the experiences of health professionals in community palliative care.

**Design:**

A qualitative Interpretive Description study was conducted with semi-structured interviews and focus groups between November 2020 and May 2021. Data was audio recorded, transcribed, and analysed using Reflexive thematic analysis. A COREQ checklist was completed.

**Setting/Participants:**

Thirty-one palliative care professionals who had participated in telehealth calls were recruited from four hospice locations in Aotearoa, New Zealand.

**Results:**

There were two themes identified: (1) ‘Getting on together’, which included how rapport shows up in telehealth, with examples of calls with rapport and without rapport, and (2) ‘Rapport is a soft skill’, which identified health professionals using body language and listening in specific ways in telehealth, while being aware of the privacy of calls, and lack of training concerns.

**Conclusion:**

Based on health professionals experiences of rapport it was determined that rapport is vitally important in telehealth calls, as it is in-person interactions. Rapport is a soft skill that can potentially be learned, practiced and mastery developed, although rapport in each interaction is not guaranteed. Patient and family experiences of rapport in the palliative telehealth area warrants further research and there is some urgency for health professional training in telehealth interpersonal skills.

**What is already known about the topic?**

- Health professionals are expected to develop rapport in telehealth interactions with patients and families.
- Telehealth has grown rapidly due to pandemic precautions, often without training and support for health professionals.
- Health professionals have concerns about developing rapport via telehealth.

**What this paper adds**

- Health professionals consider rapport is vital to telehealth calls yet find identifying rapport in interactions difficult.
- Rapport is an essential soft skill that can be learned and mastered for telehealth.
- Telehealth rapport and relational training, including managing telepresence, is needed to help health professionals adopt and integrate telehealth into palliative care.

**Implications for practice, theory or policy**

- Debriefing after difficult calls could be an important way to learn about and improve rapport soft skills.
- Developing rapport is unique in every interaction and requires a regular reflective practice to build skill.
- Examples are provided for ways that health professionals can develop rapport as a soft skill in telehealth calls.

## Introduction

The rapid introduction of telehealth into palliative care due to the Covid-19 pandemic has been disruptive to in-person interactions with patients. During times of heightened infection control precautions, in-person interactions have been restricted and replaced with telehealth calls.^1^ In a relationship-based clinical area such as palliative care, rapport is crucial during difficult and complex conversations which are often of a sensitive and existential nature.^2–5^ However, a recent review identified that there is little research concerning rapport from the viewpoint of health professionals.^6^ With telehealth becoming more commonplace in palliative care, there is a growing need for research into how well health professionals are developing rapport via digital means.

Telehealth is now considered an acceptable mode of patient contact for community palliative populations and health professionals.^7–10^ Furthermore, telehealth has the potential to improve access to palliative care and enable more equitable distribution of limited healthcare resources.^11–13^ However, palliative care professionals prefer in-person interactions^7,9^ and have expressed concerns that the remoteness of telehealth represents a barrier to care delivery and rapport building.^14,15^ Along with these concerns, health professionals want education and evidence-based telehealth guidelines to ensure a compassionate, person-centred approach to care is not lost when using telehealth.^15,16^

Although a spotlight has been on telehealth research in recent years, the topic of developing rapport during telehealth calls in community palliative care is under-investigated.^17^ While all participants are equally important in interactions that occur in telehealth, in this instance our study will focus on the health professional perspective. As the effects of the Covid-19 pandemic and future service planning are considered, it is imperative to have a better understanding of how health professionals develop rapport during telehealth interactions.^17^ Access to such information would inform the ongoing effectiveness of telehealth and its integration into palliative care.^18^ The aim of this study therefore is to gain an understanding about developing rapport during telehealth calls by exploring the experiences of health professionals in community palliative care.

## Design

This study has a symbolic interactionist lens^19^ based on the sense people make of their social worlds through social interaction, particularly through the exchange of meaning through symbols and language.^19^ We took a qualitative approach using the Interpretive Description methodology to generate knowledge for applied practice of complex experiential clinical phenomena.^20–23^ The theory of Human Relatedness^24^ underpins this study and informed the development of aspects of the interviews. For the purpose of clarity, we defined the key terms of telehealth and rapport as follows (Table 1).

**Table 1.** Key terms defined.

Telehealth	The provision of personalised health care at a distance using telecommunication means which includes video-calling and telephone.^25,26^
Rapport	A perceived connection between patient, family and health professional, which is relaxed, positive and friendly, based on caring and acceptance, with communication that is characterised by listening to and understanding of the other, to the extent the interaction fosters confidence and trust.^27^

### Population/setting

Participants were recruited from four hospices providing community services in the North and South Islands of Aotearoa, New Zealand. Participants were eligible if they were health professionals working in palliative care who had telehealth interactions with patients and families in their homes (Table 2).

**Table 2.** Inclusion/exclusion criteria.

Inclusion criteria	Palliative care health professionals from any discipline who have experience with telehealth methods of patient and family consultation.
Exclusion criteria	Health professionals with no telehealth experience. Senior management excluded as their presence may be perceived to hinder the flow of discussion.

### Sample/recruitment

We referred to the Information Power model^28^ and Interpretive Description guidelines^21^ to determine a preferred sample size of approximately 30 participants. This was anticipated to be 24 health professional participants for four focus groups and six individual interviews. A staff member at each site emailed potential participants a description of the study and invitation to participate in focus groups with consent forms attached. Potential participants self-selected, with the first six to eight respondents at each site selected to attend the focus group, no further selection process was added. Individual interviews were offered for those unavailable at the focus groups times and an appointment was made for a time and place that suited the participant. Written consent was obtained from each participant prior to research activities.

### Data Collection

Data were collected from palliative care professionals participating in focus groups or individual semi-structured interviews. A topic guide and interview schedule were developed from the literature^6,24^ asking participants for their experiences with rapport building during telehealth encounters. (See Interview guide and focus group topic guide attached). Duration of interviews and focus groups ranged between 45 and 70 min and were conducted in-person and audio recorded during November 2020–May 2021. WE, a palliative care nurse with previous experience with research interviews, conducted the interviews and focus groups and transcribed them verbatim. Memos were made after each interview or focus group. All participants were allocated a unique identifier for example, (HP 1) etc, which allowed for anonymous quotations in text.

### Data analysis

Data analysis was undertaken using a Reflexive thematic analysis^29,30^ approach as this study was interested in patterns of meaning across the data set. Our orientation to reflexive thematic analysis was experiential and as such we considered our conceptual approach to language as active and symbolic and concerned with exploring the truth(s) of participants’ experiences, perspectives and behaviours in their situated context.^30,31^

After transcriptions and field notes were loaded onto NVivo 12 software, folders were set up in NVivo to reflect the phases of reflexive thematic analysis.^29,32^ Coding started as line by line across the data set using an inductive analysis. From these codes initial themes were developed, with several subsequent themes created, reviewed and discarded, such as ‘making the call’. In reflexive thematic analysis, theme is defined as patterns of shared meaning underpinned by a central organising concept.^30^ We developed the two final themes to create insights that were relevant to addressing the research aim (Table 4).

Checklists were used to reflect on the quality and rigour of our study.^30,33^ Reflexive memos were written at each stage of the analysis which provided an audit trail for decisions made. Other decisions to promote data rigour included a reflexive journal and regular research team meetings regarding analysis.

### Ethical considerations

Consideration was given to WE being an interviewer at her place of work which was one of the four participating hospices. Provisions to ensure the safety and comfort of colleagues during the interview process included the need to disclose the dual roles of WE to participants at recruitment, and to allow participant withdrawal at any time, for any reason, until analysis began. A full Ethics proposal was granted approval by The Human and Disability Ethics committee (HDEC) New Zealand, ref: 20/CEN/165.

## Results

There were 31 palliative health professionals participants, seven participated in individual semi-structured interviews and 24 participated in four focus groups. The participants included 20 nurses, four doctors, three social workers, three family support (counselling team) and one Kaiāwhina (Māori health liaison) (Table 3). Most participants had 2+ years of specialist palliative care experience, ranging from a few months to over 30 years. All participants had phone telehealth experiences, and eight participants had experience with video calls as well. The latter included three doctors, three social workers and two nurses (Table 3). The majority of telehealth contacts were telephone calls. Video calls were mainly used to contact remote patients and families, or used as assisted calls where the specialist is at one location and the video call was set up by a nurse at the person’s home. The nature of the calls was a mixture of initial consultations and follow up calls.

**Table 3.** Participants and telehealth calls.

Professional group	Number of participants	Telephone	Phone and video calls
Registered Nurses	20	20	2
Doctor	4	4	3
Social Workers	3	3	3
Family support	3	3	0
Kaiāwhina–Māori liaison	1	1	0
Totals	31	31	8

The two themes developed during analysis were: (1) Getting on together and (2) Rapport is a soft skill (Table 4). Participant quotations have been chosen to illustrate what was interesting and important about key analytic points.^30^

**Table 4.** Key themes and sub-themes.

Themes	Sub-themes
Getting on together: How rapport was experienced by health professionals during telehealth calls.	(1) How rapport shows up in telehealth (2) Calls that went well (3) ‘Well, that didn’t go well’.
Rapport is a soft skill: Health professionals doing what it takes to develop rapport during telehealth calls.	(1) Reviewing body language (2) Listening beyond the words (3) Managing the environment (4) ‘Training would be good’.

## Getting on together

Participants perceived rapport as ‘getting on’ with each other in a way that was conducive to developing therapeutic interactions during telehealth calls. Developing rapport was central to the interactions with patients and families during telehealth calls and most participants felt it was not possible to do their jobs or make successful calls without rapport. The telehealth calls were a mix of new consultations, follow ups and fielding calls from patients or families. Some calls were in the context of established relationships over weeks or months, and some were one-off calls with no prior knowledge of the patient or family members concerned.

### How rapport shows up in telehealth

Most participants could articulate their experiences of rapport but found it difficult to report how they knew rapport was developed. However, several participants identified the indicators of rapport as non-verbal cues such as tone of voice, flow of speech and speed of speaking which changed when rapport was beginning to develop. For example:

*I think it feels good, cause you have established a relationship and there is that flow of information and you just hear them relax a bit more. (HP 1, phone call)*

Other participants noticed a shift in the quality and content of the conversation and some participants spoke of feeling a sense of mutuality in the interactions. For instance, one participant describes reaching a point of being comfortable with each other.

*And to me, it’s that common ground but also making people comfortable because whether it’s face to face or phone or video, if you are not comfortable with each other, then nothing is going to happen, nothing of importance. But if you don’t have that rapport, you can’t get them to open up to you. (HP B)*

For other participants there was no clear indication of mutual rapport developed but they perceived a connection personally and felt the interaction was positive. Several participants made the statement that it was easier to discern if rapport was established during in-person interactions rather than in phone calls due to the lack of visual cues on the phone.

### Calls that went well

Most participants felt they had positive and effective experiences of developing rapport during telehealth calls most of the time. One participant described the rapport she had with one of her longer term patients was better on the phone call than previous in-person visits.

*I got lots of what I would call information that I hadn’t had before, because he (patient) felt comfortable talking to me on the phone. . . . I was thinking on the phone in that conversation, oh my god, that’s the first time he has really mentioned that. (HP E-phone call)*

The eight participants who tried video calls all had positive reports about rapport building and were keen to carry on with video calling in their practice, with comments like ‘Completely converted!’ and ‘Sometimes more comfortable than being in the room’. One participant preferred video to phone calls.

*I found I preferred a video chat to a telephone. To be able to see her and to make comment on the things that I would notice that were different, because for her that was really actually quite important. It did enhance our relationship. (HP 21- video call)*

### Well, that didn’t go well

However, as well as many positive telehealth calls, most participants also recounted calls that were complex and challenging with no rapport developed, all of which were phone calls. Despite the challenges with some patients and families via telehealth, participants felt they needed to persevere to form some sort of rapport and relationship.

*On the phone, quite different because she couldn’t see me, she could only hear me. It’s like she didn’t trust anything that I said. I was not comfortable at all, and I just kept thinking, I need to keep the engagement up, I need to keep her, you know, talking, confident, sharing, whatever, and it really was a struggle. (HP E- phone call)*

Participants had calls where they had to manage strong emotions like anger and frustration from patients and families. Sometimes rapport was abandoned to be able to safely end the call, for example:

*Yeah, and I know when I’m working hard because I’m more calculated with my words and I think my speech slows down. It’s because I am thinking about every word that I’m saying in case it can be mis-interpreted or used as ammunition back. So I’m thinking of someone that is angry on the phone. I’m very calculated, I can hear myself and I can see myself really slowing down and having pauses. And less personality from me. It’s a challenge to think, can I turn this situation around. How quickly can I do it? If I can’t do it, then, it’s not alright but I have to think of a way to establish a relationship if it’s not going well. (HP F- phone call)*

Three experienced participants had the distressing experience of being hung up on during a telehealth phone call. Rapport was not developed during these calls, as one participant described:

*Yeah. Hung-up-on-me. It is hard when someone hangs up on you. It is hard because. . . I always like to finish off a phone call on as good a term as possible and usually there is some agreement or there’s some plan. . .And then someone hangs up on you, it’s just left in the air and you are thinking, well clearly, she is upset, she’s not happy. Things haven’t ended in a good space. (HP D- phone call).*

## Rapport is a soft skill

Despite the challenging calls, most participants demonstrated their desire and ability to do what it takes to develop rapport during telehealth calls. Participants felt it was their responsibility to ensure rapport was developed as it was vitally important to have a safe connection and trust to begin working together. Due to the determined approach health professionals had towards developing rapport we interpreted that participants were developing and managing rapport as a soft skill during telehealth calls. The concept of soft skills is defined here as personal and interpersonal skills that contribute to productive and harmonious relations between health professionals and patients and families.^34^

### Reviewing body language

Participants were aware that their body language was different in telehealth. Participants felt more aware of their facial expressions, eye, head and torso movements. Participants were sometimes unconscious at the time they were adjusting their body language to develop rapport.

*It [video] feels very comfortable. I think probably initially when we first started doing it there is always a bedding in process and it takes a while to get used to it, but now it feels almost second nature. Almost the same as doing a face to face conversation really. You can’t use the same body language, it’s basically just your face on the screen so you know maybe, and I don’t know whether I would do this purposefully, but maybe you have to use more facial expression rather than relying on your body language to kind of gain trust and build rapport. (HP A- video call)*

As well as awareness of their own facial movements during the video calls, participants were checking the facial expressions of patients or family on the video screen to assess for signs of rapport.

*And I could see that because that’s the joy of a video, you can see people’s expression and when it was not a good connect (HP E-video call)*

### Listening beyond the words

The key communication skill identified by participants to develop and manage rapport was that of listening. Most participants said they actively listened, read between the lines or were listening for what was not said. Listening to understand during a telehealth call enabled the health professional to interpret what was said beyond the spoken words. For one participant the way to develop rapport during challenging calls was to listen and to prompt the family member to talk until they had a sense of being heard.

*So they sound, that same thing, that tone changes. They don’t sound so irate. They probably feel more listened to and heard and it’s just that someone is actually helping them. Yeah, someone is doing something to help. (HP B, phone call)*

Listening was a gateway to developing rapport and building trust with the person. Some participants understood listening to be personal, and sensitive to nuances in speech, tone and silences during telehealth calls.

*Because you are listening. You don’t have a distraction; you are listening, and you can pick up the nuances and you can hone in. HP E.*

### Managing environment and privacy

Participants were aware that telehealth required private space to develop rapport. However most work environments were not set up for telehealth at all. There was often a lack of private space to use video or phones in busy clinical areas and shared offices. Health professionals often moved to try and find quiet rooms to make telehealth calls that would allow uninterrupted flow and concentration.

*Often if our office is busy or noisy it can be hard to concentrate. Like the environment that you have made the phone call in can actually affect it sometimes. You might want to end that phone call sooner because you can’t quite hear or get a sense or concentrate. It doesn’t happen often, but it can affect how you do your phone call. So sometimes I have to walk, I walk away and go into an office and make a phone call there and have a chat to them. (HP G Phone calls)*

Working from home during the pandemic also posed privacy issues with one participant going to the extent of closing her windows so the neighbours couldn’t hear her client’s conversations.

### ‘Training would be good’

Several participants pointed out the lack of training in telehealth interpersonal relationship building, which they felt would have been beneficial for themselves and patients. Due to a lack of training and practice opportunities some participants voiced a hesitancy to offer video telehealth calls. One participant had a sense of ‘wasting time’ trying to sort out technology for video calling without having tried it, and felt her own uncertainty was a risk to vital first impressions and creating rapport.

## Discussion

This study is the first of its kind to explore palliative care professionals experiences of rapport during telehealth calls. Participants considered rapport to be essential during telehealth interactions, just as it is in face-to face visits. Importantly participants felt they developed rapport most of the time, but they also identified times when rapport was not achieved despite their best efforts and expertise. Some participants found it difficult to articulate when or how rapport was experienced during a telehealth interaction. Video calls were positive with rapport developed, whereas the most distressing telehealth calls were phone calls. In spite of this, participants used the phone as the ‘go to’ for telehealth. A key result of our analysis is that developing and managing rapport is a soft skill that is essential to in-person and telehealth interactions and yet overlooked in terms of training.

Health professionals are more likely to use technology when they are trained and supported in how to use telehealth in their practice.^11,35,36^ Unfortunately, due to the Covid pandemic, many health professionals were not provided with a training opportunity to develop skills to navigate rapport in the telehealth environment.^37–39^ As in other studies, our findings suggest that there is a gap in professional preparation for telehealth.^40^ In this study, participants were not asked explicitly how much telehealth training they had, but most indicated telehealth was approached by trial and error, while recognising a need for interpersonal skills training. Some untrained participants in other studies were also tentative to try video calls as they felt video may interfere with vital first impressions and rapport, as the technology might fail, or waste valuable relationship building time.^13,35^

The ‘home visit’ context of community palliative care is mostly soft skills based, whereas telehealth is a mix of ‘hard’ technical skills and ‘soft’ communication skills, such as developing rapport remotely which requires focus, intention and practice.^27,41^ To adapt to telehealth, health professionals need to consider how they can transition their in-person rapport skills while maintaining technology and patient-centred care in telehealth.^42^ A recent Swedish study gave an example of merging hard and soft skills during telehealth calls, when some nurses and patients simultaneously placed their palms on their respective screens to create a sense of proximity. These nurses had participated in online telehealth training which boosted their theoretical knowledge confidence and competence.^43^

The concept of soft skills is a powerful way to conceptualise rapport as it implies rapport can be practised and developed into mastery in contexts like telehealth.^44^ This applies equally to health professionals understanding telepresence without which rapport could not be realistically experienced.^37,45,46^ Even with mastery though, rapport is not guaranteed, and requires attention in every interaction. Rapport as a soft skill mitigates the common belief that rapport just happens, or that rapport is personality dependent.^38,47^ Although more research is needed into rapport and telehealth, researchers have identified the qualities required for successful rapport building and developed a checklist for telehealth skills.^44,48^ Further research is also warranted to reflect the vital experiences that patients and families have of rapport in telehealth.

### Limitations of the study

We noted few focus groups participants had video-call experiences, whereas most interview participants had video-call experiences, this indicated difference responses to recruitment to the two options. Data from the interviews also provided richer examples of rapport experiences. Focus groups may have limited the freedom to describe the individuals experiences of rapport and were difficult to recruit and plan for during the pandemic. However, the interpretive design allowed for exploration of the themes to generate knowledge about rapport that can be applied in clinical practice.

Despite efforts to recruit a diverse population of participants, most participants were New Zealand European and therefore may not reflect different cultural approaches to telehealth. Although conducted in the context of palliative care the results of this study may be generalisable and prove useful to those interested in telehealth and developing rapport with patients and families in their homes.

### Implications

Developing rapport is a foundational soft skill for health professionals and is suitable for a regular reflective practice to learn from telehealth interactions.^27^ To demonstrate the skills required to develop rapport during telehealth, examples are provided from recent telehealth studies that focused on interpersonal skills development (Table 5). The categories are drawn from the key components of the definition of rapport^27^ and the studies referenced.^4,15,43,48–51^

**Table 5.** Developing rapport as a soft skill in telehealth calls.

Category	Examples of actions or approaches to develop rapport	References
Preparation	- Check technology so that you can see and hear each other. - Check also for non-technological barriers such as the person’s level of hearing and their preferred mode of communication.	Banerjee et al.^49^ Webb et al.^15^
Presence/Telepresence	- Give each call your full attention and focus on the patient and family. Trying to do something else at the same time is very obvious on the phone or video call. - Pay attention to nonverbal communication (self and other) - If on a video call, make appropriate eye contact by looking at the camera.	Schrager^50^ Carlsson et al.^43^ Webb et al.^15^ Watts et al.^4^
Privacy	- Confirm your patient is in a setting where they feel comfortable discussing their private health information. - Determine how the information discussed in the call is made available to the patient	Banerjee et al.^49^ Watts et al.^4^
Clarity	- Speak slowly and clearly - Avoid medical jargon - Clarify unclear statements	Webb et al.^15^ Schrager^50^
Relaxed positive friendly	- Introduce yourself and your role, offering a warm welcome - Express positive views of telehealth - Smile and use a friendly tone of voice - Open posture, lean forward, relax and focus - Spend time to get to know the person as a person at the beginning of the call, use ‘local’ references.	Henry et al.^48^ Webb et al.^15^ Watts et al.^4^
Acceptance	- Use culturally appropriate greetings and show respectful awareness and acknowledgement of whanau/family in the room. - Check if the patient would like a support person to be with them, or if they need an interpreter - Endorse and encourage patient and families to ask questions or take notes - State that you aim to work as partners and listen throughout for ways to work together.	Hilty et al.^51^ Watts et al.^4^ Banerjee et al.^49^ Henry et al.^48^
Caring	- Use caring words, sounds and tone of voice - Check in on persons comfort and emotional wellbeing - Make a caring statement expressing that the patient’s emotional response to an event or an experience is appropriate and reasonable.	Webb et al.^15^ Banerjee et al.^49^
Listening	- Avoid interruptions – Pause after speaking and wait a few seconds before responding to a patient to reduce interruptions. On video use visual cues, such as nodding, or positive verbal utterances to show that you are listening - Listen actively and do not make assumptions about what you have heard. Ask ‘Have I heard you correctly?’	Henry et al.^48^ Banerjee et al.^49^
Understanding	- Seek clarification for what you have said, ‘I would like to hear from you, what do you understand from our discussion so far?’ - Reflect your understanding of what the patient has said. ‘So I want to make sure I am understanding what you are saying. . .’	Schrager^50^
Clarify next steps	- Go over main points of your discussion and encourage questions. - Establish what will happen after you hang up. If you have the capability, send an after visit summary through a portal so the patient will have something in writing from the phone visit.	Banerjee et al.^49^ Schrager^50^

## Conclusion

Health professionals felt they developed rapport most of the time using telehealth, while also identifying times when rapport was not achieved despite their best efforts and expertise, particularly on the phone. Importantly, rapport is identified as a key soft skill that can be practiced, and mastery developed, although rapport in each interaction is not guaranteed. There is some urgency for health professional training to improve integration of telehealth into palliative care. Studies that include patient and family perspectives on rapport in the palliative care and telehealth contexts would be a promising area for further research.

## Supplemental Material

sj-pdf-1-pmj-10.1177_02692163231172243 – Supplemental material for Health professionals’ experiences of rapport during telehealth encounters in community palliative care: An interpretive description study Click here for additional data file. Supplemental material, sj-pdf-1-pmj-10.1177_02692163231172243 for Health professionals’ experiences of rapport during telehealth encounters in community palliative care: An interpretive description study by Wendy English, Jackie Robinson and Merryn Gott in Palliative Medicine

sj-pdf-2-pmj-10.1177_02692163231172243 – Supplemental material for Health professionals’ experiences of rapport during telehealth encounters in community palliative care: An interpretive description study Click here for additional data file. Supplemental material, sj-pdf-2-pmj-10.1177_02692163231172243 for Health professionals’ experiences of rapport during telehealth encounters in community palliative care: An interpretive description study by Wendy English, Jackie Robinson and Merryn Gott in Palliative Medicine
