# Supplementary material for: Health professionals’ experiences of rapport during telehealth encounters in community palliative care: An interpretive description study
Source: Palliat Med. 2023 May 2;37(7):975–83. doi: 10.1177/02692163231172243 (PMC10320708; doi:10.1177/02692163231172243)
Supplement: sj-pdf-1-pmj-10.1177_02692163231172243 – Supplemental material for Health professionals’ experiences of rapport during telehealth encounters in community palliative care: An interpretive description study [file sj-pdf-1-pmj-10.1177_02692163231172243.pdf]

Focus group topic guide:

Welcome and thank you for participating in this focus group.

Introduce moderator and ground rules:

Introduce topic: This focus group discussion is designed to assess your thoughts and feelings about telehealth in palliative care. It aims to explore both your experiences and some of the early feedback from the patient and family interviews about their experiences with telehealth calls. For this project telehealth is defined as telephone or video calls made between health professionals and patients or family/whānau receiving palliative care at home.

Recorder on, asking participants to introduce themselves. Name, profession, how long practicing, how long in palliative care?

Questions:

1. Take a couple of minutes to think about your experiences of phoning or video calling patients or family members at home. Is anyone happy to share their experience? (10 minutes).
   What went well?
   What did not go well?
   Vs Face-to-face
   Lockdown
   Meeting prior to calls
   After hours calls
2. Patient feedback (20 minutes). Detailed below.
3. As you look to the future how do you see telehealth fitting in with palliative care? (10 minutes)
   Professionally
   Your practice
   Patients
   Whānau

Summing up:

Of all the things discussed here what do you consider the most important? (5 minutes)

Is this an adequate summary?

Do I have everything covered?

Thank you. Deep gratitude for the gifts you have given to our professions.
Nga mihi nui
Tena kotou, tena koutou, tena koutou katoa.

Recorder off.

Preliminary patient and whānau feedback from interviews

Theme 1: Not understanding the system

- Who's the best person to call and when?
- How do I find out… if I don't know that I don't know?

**Soundbites:**
I don't know who to call first, the DN the GP or the specialist team<br>I know I can ring hospice anytime<br>I wait till I really need to call the hospice<br>I need a case manager, one person to deal with<br>I have become a coordinator of services and don't know what I am doing<br>I bought the equipment and then found out we could borrow it

Theme 2: Speaking up and stepping up

- Patients and family members have to learn new communication skills to get needs met by health professionals
- Role change for partners
- Barriers can be: not understanding accents, Hearing loss, Brain fog

**Soundbites:**
I don't want to waste your time when I ring<br>I should have said that I didn't understand and had never done it before (given subcut pushes-Family member)<br>I make up half of it and feel anxious that I got it wrong (hearing loss, medication effects)<br>I was told I was "wasting resources"<br>I am not important<br>I knew the CNS had spoken to hospice about my husband, they knew my name when I rang

**Rapport means**	**A lack of rapport means**
Feeling heard<br>Feeling seen<br>I am involved<br>I am comfortable and can say anything<br>I trust what is being said<br>I feel supported	Feeling frustrated<br>Feeling stupid<br>I am not involved<br>I am not comfortable<br>There is no trust<br>I feel unsupported
