# Supplementary material for: Health professionals’ experiences of rapport during telehealth encounters in community palliative care: An interpretive description study
Source: Palliat Med. 2023 May 2;37(7):975–83. doi: 10.1177/02692163231172243 (PMC10320708; doi:10.1177/02692163231172243)
Supplement: sj-pdf-2-pmj-10.1177_02692163231172243 – Supplemental material for Health professionals’ experiences of rapport during telehealth encounters in community palliative care: An interpretive description study [file sj-pdf-2-pmj-10.1177_02692163231172243.pdf]

# Health Professional Interview Schedule:

Thank you for you your time in participating in this interview

I am going to ask a few questions about your experiences with and thoughts about phone or video calls with patients and families at home.

But first tell me how long you have been practicing? and how long in palliative care?

1. How often would you make phone or video calls to patients and families as part of your average day? Week?
2. What kind of calls are these?
3. Do you initiate them or do Patient and families initiate them?
4. Do you make video calls? Did you during Lockdown periods? What was your experience of these?
5. Take a moment to think about some of the phone or video calls you have made generally:
    - Can you think of a time when a call with a patient or family member has gone really well? What is it about that call that went well? How was that for you?
    - How would you describe the call in terms of comfort for you? How comfortable were you?
    - How would you describe the call in terms of involvement for you? How involved were you?
    - What if anything is important about that for you?
    - Tell me more about that
6. Again thinking of the calls with patients and families can you think of a time(s) when the call did not go so well? What is it about the call that did not go well? How was that for you?
    - How would you describe the call in terms of comfort for you? How comfortable were you?
    - How would you describe the call in terms of involvement for you? How involved were you?
    - What if anything is important about that for you?
    - Tell me more about that?

7. I am interested in the idea of rapport and especially what you understand it to mean? How would you describe rapport? What other types of words would you use to describe rapport?

- What if anything is important about rapport?

8. What kind of things might you say to (their words) create rapport with patients/whanau?
    - What kinds of things might you do?
    - Do you have an example(s) of that working?
9. How do you know when you have (created rapport) with the other person? What is an example of that?
    - How does that feel for you?
10. What about when rapport is not created? What is an example of that?
    - How does that feel for you?

11. How does the use of telephone or video affect how you create rapport with patients and whanau? What if anything is different about your rapport building?
12. If you have not used video calling for clients, what would you need to make you feel more likely to undertake a call this way?

13. If you look to the future, how do you see telehealth calls fitting into palliative care?
    Professionally?
    Personally?
    Patients?
    Whanau?
14. Thank you. Before we finish do you have anything else you would like to say?

Nga mihi nui. Thank you so much.
